# Real-Time Adaptive Traffic Signal Control in a Connected and Automated Vehicle Environment: Optimisation of Signal Planning with Reinforcement Learning under Vehicle Speed Guidance

**DOI:** 10.3390/s22197501

**Published:** 2022-10-03

**Authors:** Saeed Maadi, Sebastian Stein, Jinhyun Hong, Roderick Murray-Smith

**Affiliations:** 1 Urban Big Data Centre, Department of Urban Studies, University of Glasgow, Glasgow G12 8QQ, UK; 2 School of Engineering, Damghan University, Damghan 36716-41167, Iran; 3 School of Computing Science, University of Glasgow, Glasgow G12 8QQ, UK; 4 Smart City Department, University of Seoul, Seoul 02504, Korea

**Keywords:** connected and automated vehicles, adaptive traffic signal control, reinforcement learning, microscopic traffic simulation

## Abstract

Adaptive traffic signal control (ATSC) is an effective method to reduce traffic congestion in modern urban areas. Many studies adopted various approaches to adjust traffic signal plans according to real-time traffic in response to demand fluctuations to improve urban network performance (e.g., minimise delay). Recently, learning-based methods such as reinforcement learning (RL) have achieved promising results in signal plan optimisation. However, adopting these self-learning techniques in future traffic environments in the presence of connected and automated vehicles (CAVs) remains largely an open challenge. This study develops a real-time RL-based adaptive traffic signal control that optimises a signal plan to minimise the total queue length while allowing the CAVs to adjust their speed based on a fixed timing strategy to decrease total stop delays. The highlight of this work is combining a speed guidance system with a reinforcement learning-based traffic signal control. Two different performance measures are implemented to minimise total queue length and total stop delays. Results indicate that the proposed method outperforms a fixed timing plan (with optimal speed advisory in a CAV environment) and traditional actuated control, in terms of average stop delay of vehicle and queue length, particularly under saturated and oversaturated conditions.

## 1. Introduction

Traffic congestion has been a key urban issue, causing high economic costs in many cities worldwide. The report carried out by the Institute of Economic Affairs (IEA) claims that just a two-minute delay to every car journey costs the economy approximately 16 billion GBP a year, or nearly one percent of GDP (gross domestic product) in the UK [1]. Traffic lights play an essential role in controlling traffic flow and minimising delay, especially in an urban area. As a result, the number of traffic lights in England has increased by 25% since 2000, while the number of cars on roads grew by just 5% [1]. However, most of them use offline control systems based on the historical traffic flow and cannot respond to unexpected traffic situations (e.g., car accidents) efficiently or predict future traffic flows. Thus, adaptive traffic signal control (ATSC) approaches have been developed by numerous researchers [2,3,4]. Recently, advancements in computer performance and new optimisation methods have allowed researchers and practitioners to adopt heuristic methods for the ATSC process (e.g., real-time hierarchical optimising distributed effective system, RHODES [5]). These systems try to optimise traffic signal parameters in real time without considering a cyclic time interval. Thus, the signal plan could change at any time step depending on rapidly changing traffic conditions [6].

In recent years, autonomous vehicles (AVs) and connected vehicles (CVs) have been introduced due to the new generation of automation and connectivity technologies. Reference [7] reported that AVs could save the US economy roughly 211 billion USD annually with a 50% AV market penetration rate. They also suggested that the combination of automation and vehicle-to-everything (V2X) communication (vehicle-to-vehicle (V2V) and vehicle-to-infrastructure (V2I) communication) is called connected and autonomous vehicles (CAVs), and they could further reduce congestion and fuel consumption. These will not only be an opportunity for transport planners to improve current capacity utilisation but will also be a research challenge for them in order to adapt the current transport systems to this new traffic environment [8]. As CAVs are gradually developed, they would also call for further research on intelligent signal control in the near future traffic environment [9]. Considering CAV technology, vehicles and traffic infrastructure can communicate with each other. For example, sending real-time trajectory data on speed, location and headway from CAVs to signal controls enables planners to optimise better traffic signal control plans based on accurate current and future traffic flow predictions [10,11,12,13,14]. In addition, CAVs can drive more efficiently by controlling vehicle speed and acceleration in response to signal timing plans, further improving the overall network performance (reducing the number of stops, fuel/energy consumption, and emission) [15,16]. Unfortunately, most existing CAV studies assumed fixed traffic signal timings to optimise the trajectory of CAVs [17,18,19,20].

Learning-based methods such as reinforcement learning (RL) have achieved promising results in lane-change behaviour under CAVs environment with self-learning and high-efficiency calculation [21]. In this work, we use RL in signal plan optimisation under the CAVs environment. In an RL approach, the agent (vehicles, traffic signal control) learns from the state (traffic environment) by taking actions (e.g., lateral control, signal plan changing) and observing the feedback rewards (e.g., lane changing time consumed, queue length). That can improve the lane-change manoeuvres or optimise the signal plan. In the signal plan case, agents learn by replicating traffic signal plans in a closed-loop system, leading to a model-free, self-learning framework [22] that can adapt to network-wide real-time traffic changes.

This study adopted an RL-based approach to consider both the optimisation of traffic signal control plans and CAV environments, given the above motivations and context. The highlight of this work is the speed guidance algorithm that helps to reduce the stopped delays of vehicles. The idea of combining a speed guidance algorithm with a reinforcement learning-based traffic signal control system is new in this area. In our RL approach, the intersection, as an agent, adopts traffic signal plans to optimise the total queue length. Simultaneously, the CAVs adjust their speed based on a fixed timing plan to decrease total stop delays, which are the waiting time for a vehicle when its speed is zero (practically when its speed is less than 5 mph) [23]. As a result, the agent adapts the traffic signal control to an adjusted speed of CAVs in real-time (updated traffic flow). Based on this framework, two different performance measures—minimising total queue length and total stop delays—are combined into a performance index (PI). The signal controller (agent) was trained in the fully dynamic traffic environment (traffic flows and CAV speeds) under different demand levels (unsaturated, saturated, oversaturated) and CAV penetration rate scenarios (0%, 25%, 50%, 75%, 100%) to show potential interaction effects between signal plan, CAV penetration rate and traffic flow. Results indicate that the proposed approach outperforms a fixed timing plan as well as conventional vehicle actuated control in terms of the average stop delay of vehicles and queue length (performance index). This work uses a microsimulation model in the VisVAP module of the Vissim 20 program for actuated control that is based on the guidelines of the Federal Highway Administration (FHWA) [24]. The actuated control prioritises the phase with the primary approach (main road), and detector actuation partially controls each phase’s time.

The rest of this paper is organised as follows. Section 2 presents the relevant literature review. Section 3 presents our integrated reinforcement learning (RL) and speed guidance approach under the CAVs environment. In Section 4, the simulation setup is presented, followed by our evaluation results in VISSIM microsimulation software. Finally, in Section 5, we conclude and provide ideas for future work.

## 2. Related Work

**Figure 1:**
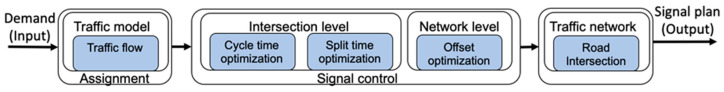
Open-loop traffic signal control system.

Adaptive traffic signal control approaches, including SCOOT [25] and SCATS [26], have been widely used in real-world traffic networks. They are based on an open-loop control system that does not consider feedback control in the traffic network (Figure 1). They use a cyclic system with pre-determined time intervals, which means the controller updates the signal timing plan (cycle length, green signal ratio, and phase difference) at a specific time interval [27]. Studies show that the traffic flow at intersections may vary significantly in major cities due to the fluctuation of traffic demand [28,29]. Nevertheless, these typical adaptive traffic signal control systems cannot respond to such travel demands (traffic flow varies at shorter time intervals) [6] and require complex computation schemes that make their implementation costly [30]. This can increase travel delays for road users.

As mentioned in the introduction section, adaptive traffic signal control in a connected vehicle environment has shown a positive effect on the improvement in network efficiency [10,11,12,13]. Connected vehicle technology is a mobile data platform that allows real-time information to be exchanged among vehicles and between vehicles and infrastructure [31].

### 2.1. Traffic Signal Control under CAVs

As mentioned in [15], there are many novel approaches for CAV-based traffic control. One of the most common methods, which can be seen in various studies, is the ‘advanced driver guidance’. In this approach, vehicle speeds and positions are adjusted to minimise some performance measures. Reference [32] proposed an integrated traffic control model to optimise the total delay, and the decision variables for this research were vehicle arrival time and signal timing. They simulated the speed guidance model of CVs in VISSIM microsimulation software. They concluded that this method could significantly decrease vehicle delays and the number of stops. Reference [33] introduced GLOSA and assessed its benefits in reducing vehicles’ stop time behind a traffic light and fuel consumption using an integrated cooperative ITS simulation platform. Under two simulated scenarios, reference [34] investigated the positive impacts of the speed guidance on fuel consumption and driving behaviour for multiple signalised intersections. Other methods can be found in the literature, for instance, planning-based traffic signal control [35], platoon-based traffic signal control [36], and signal vehicle coupled control (SVCC) [15,37]. Unfortunately, most existing CAV studies assumed fixed traffic signal timings to optimise the trajectory of CAVs [18]. Unlike the current research methods, this paper presents an adaptive traffic signal control under the CAVs with the advanced traffic control (speed guidance model).

### 2.2. Closed-Loop Signal Control

Compared to typical adaptive traffic signal control (open-loop) systems, two general closed-loop signal control approaches exist. The traditional signal control approaches (i.e., non-learning-based approach) use a simple feedback loop control system and utilise only the current traffic flow but not historical traffic flow data. In addition, these approaches do not have underlying models for state prediction and optimisation. A learning-based method such as reinforcement learning can learn from the traffic environment by taking actions (i.e., cycle length and phase split) and observing the feedback that can help us predict the traffic flow and optimise the signal plan [32].

#### 2.2.1. Non-Learning-Based Approach

Some studies propose a simple closed-loop system (non-learning-based approach) in traffic signal control. Reference [38] presented a decentralised feedback control mechanism aiming to equalise the degree of saturation and queue length on different approaches toward intersections in a network. Reference [39] showed a localised feedback speed control for mainline traffic on motorways. The backpressure controller is a distributed feedback system that does not require knowledge of global network inflow. Reference [40] introduced the traffic-responsive urban control (TUC) strategy as a network-wide feedback approach. Based on store-and-forward modelling of the urban network traffic and using the linear-quadratic regulator theory, the design of TUC leads to a multivariable regulator for traffic-responsive coordinated network-wide signal control that is also particularly suitable for saturated traffic conditions. Reference [6] proposed an upgrade closed-loop feedback signal control strategy, which takes the total amount of instantaneous stopped delay (ISD Total) as input detector data for measurement frame-by-frame in traffic flow video and realised real-time switches of the signal status when the amount reaches the threshold, and adaptively distributes the green interval to the most needed approaches (east–west and north–south) without the regular traffic signal cycle time. In other words, they still used a non-learning-based method but with new forms of data.

#### 2.2.2. Learning-Based Approach (Reinforcement Learning)

Reinforcement learning-based adaptive traffic signal control changes traffic signals based on the feedback from the traffic demand, which can be hypothetical dynamic [41,42,43,44] or based on real-world data [45,46]. The existing literature on using the reinforcement learning approach can be categorised into two groups: networks consisting of CVs [44,47,48] and non-CVs environments [45,46,49]. Moreover, two general classifications (i.e., vehicle positions and queue length) are available for state representation. References [41,43,49,50] proposed the state as discrete values such as the position of vehicles or different levels of queue length. Nevertheless, this kind of state needs massive storage space for solving large problems. Therefore, recent studies recommend the use of continuous states such as queue length [42,45,51], average delay [44], and waiting time [30,42,48]. Furthermore, all of the papers relevant to this topic have used simulation platforms in order to obtain their desired results. SUMO [43,44,49,50], VISSIM [46,47], AIMSUN [45], and PARAMICS [30] are the most common software packages in which the combination of traffic simulation and RL can be executed appropriately. Finally, it is worth mentioning that all the previous papers took the signal controller as an agent for their RL algorithm except [47], which used connected vehicles as its agents. 

This research aims to bridge the gap between the RL-based adaptive traffic signal control and the advanced traffic control (speed guidance model) under CAVs. The RL based approach focuses on the intersection as an agent in order to optimise total queue length while allowing the CAVs to adjust their speed based on a fixed timing plan to minimise the total stop delays as an agent adapts the traffic signal control to an adjusted speed of CAVs in real-time (updated traffic flow).

## 3. Methods

This section discusses the proposed framework for RL, PTV VISSIM microsimulation platform and implementation details. The RL framework is implemented in Python and integrated into VISSIM through the component object model (COM) interface (COM interface of PTV VISSIM).

### 3.1. Reinforcement Learning (RL)

RL is an area of adaptive control that encompasses algorithms for learning optimal behaviour policies in sequential decision-making problems from scalar rewards. It is assumed that a decision-making problem is time-discrete, and it can be represented with a Markov decision process (MDP) (*S*, *A*, *P_a_*, *R_a_*), where S is the state space, A is the space of possible actions, *P_a_*(*s_t_*, *s_t+_*_1_) = *P*(*s_t+_*_1_*|s_t_, a_t_*) is the probability of transitioning into state *s_t_*_+1_ by taking action *a_t_* in-state *s_t_*, and *R_a_*(*s_t_*, *s_t+_*_1_) is the immediate reward received after transitioning *s_t_* into *s_t+_*_1_ by taking action *a_t_*. The agent is specified by a policy π(*a*|*s*) mapping each state to a probability distribution over actions. An optimal policy maximises discounted return *G_t_*_=0_, which is defined as the sum of discounted future rewards (see (1)). The state-value function *V*_π_(*s*) is the expected return of a state *s*, assuming the agent follows policy π in all time steps (see (2)).

*G_t_* = *R_t_*_+1_ + γ*R_t_*_+2_ + γ^2^*R_t_*_+3_ + ... = *R_t_*_+1_ + γ*G_t_*_+1_ (1)

*V*_π_(*s*) = 𝔼*_a_*_~__π_ [*P_a_*(*s*, *s*^′^) *R_a_*(*s*, *s*^′^) + γ*V*_π_(*s*^′^)] (2)

Model-based RL algorithms assume *P_a_* and *R_a_* to be known, while model-free RL algorithms learn about them implicitly from the interaction between agents and the MDP. This paper uses the advantage actor-critic algorithm (A2C), a model-free RL algorithm suitable for stochastic environments with partially observed states. The policy *π*(*a|s*; *θ*) and state-value function *V*(*s*; *θ_v_*) are approximated by neural networks with parameters *θ* and *θ_v_*, respectively. Initialised at random, the policy is optimised through iterative policy evaluation (gathering experience by interacting with the environment using the current policy) and policy improvement (reinforcing actions that led to greater than expected rewards and discouraging others) by gradient descent using (3) and (4).

d*θ* = ∇log *π*(*a*|*s*; *θ*) (*G_t_* − *V*(*s_t_*; *θ_v_*)) (3)

d*θ_v_* = ∂/(∂*θ_v_*) (*G_t_* − *V*(*s_t_*; *θ_v_*))2 (4)

In order to apply A2C to traffic signal control, we need to define the state space S and the action space A. Monte Carlo samples of *P_a_*(*s*, *s*′) and *R_a_*(*s*, *s*′) are generated by applying the agent policy in an episodic microscopic traffic simulation. We defined the action space of an agent that controls a single intersection as the set of possible signal phases. In this paper, all intersections have four approaches controlled in three phases. At every decision time step, the agent selects the next signal phase. An inter-stage is executed (switching the active signal group to red and the selected signal group to green) if required, and the chosen phase is applied for a minimum duration before the agent receives its immediate reward and the subsequent environment state. The state is represented as the concatenation of the following features:

- a vector encoding the current queue lengths on all incoming lanes,
- a one-hot vector encoding of the last chosen signal phase at time *t* − 1,
- the elapsed time since the last signal phase change,
- for each signal phase, the elapsed time since it was last active.

Elapsed time here is measured from the agent’s perspective in the number of decision time steps. The queue lengths along all approaches represent a partial observation of current traffic demand, and all other state information summarises aspects of recent signalling history. The latter allows the agent to anticipate demand from elapsed time or learn a deterministic signal program (if that was an optimal solution). The instantaneous reward *R_a_*(*s*, *s*′) is defined as the difference between the average queue length across all incoming lanes in state s and the average queue length in state *s*′. Thus, a positive instantaneous reward is given if the average queue length after an action is reduced and vice versa. We also explored using average vehicle delay as immediate rewards in preliminary experiments but discovered a simulation artifact that resulted in reward hacking. As the vehicle delay in PTV VISSIM is only counted after a vehicle passes the intersection, agents would converge to suboptimal policies that prevented vehicles from crossing the intersection to avoid the associated penalty. The policy network *π(s; θ)* consisted of three dense hidden layers with 64 units each and leaky ReLU activations (α = 0.05), followed by a Softmax layer with one unit per signal phase. The value network *V(s; θ_v_)* also consisted of three dense hidden layers with 64 units each and leaky ReLU activations, followed by a single-unit linear layer. The state was normalised to [0, 1] independently along each dimension before it was fed into these networks. During training, the agent gathered experience by interacting simultaneously with two environment instances, which has been shown to stabilise training by de-correlating batches of experience. We used the Adam optimiser with a learning rate 1 × 10^−4^, gradient clipping at value 1.0, and entropy regularisation. The contributions of the policy loss, value loss and entropy loss were weighted with weights *w_π_* = 1.0, *w_v_* = 0.5, and *w_h_* = 0.01, respectively. The agent was able to choose a signal phase once every 5 s of green time (ignoring the time spent changing signal phases). With this configuration, all agents were trained for 100 simulation episodes, each simulating two hours of traffic. 

### 3.2. Simulation Platform

**Figure 2:**
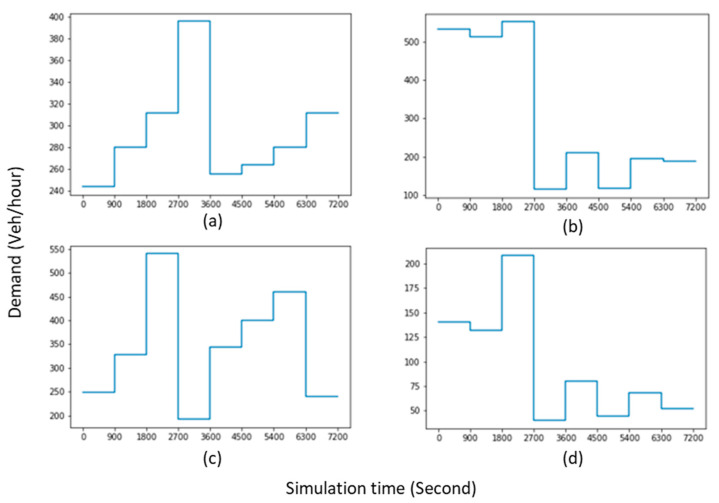
Time-varying arrival rates at 15-min interval. (**a**) East entry; (**b**) West entry; (**c**) South entry; (**d**) North entry.

In this paper, PTV VISSIM microsimulation software was used to investigate the impact of the proposed framework on performance measures such as queue length and stop delay. Therefore, akin to some relevant studies [47,52], an isolated four-leg signalised intersection was employed in this study (north entry has one line, whereas other approaches have two lines). This intersection has been taken from a sample example in VISSIM (used as a template to show the benefits of three-stage vehicle actuated signal control over fixed time) to represent the functionality of the proposed method in dynamic traffic demand. As shown in Figure 2, time-varying arrival rates were generated based on the VISSIM example at 15-min intervals for two hours to consider the fluctuation of traffic demand in a real-world network.

### 3.3. Driving Behaviours

In this study, two vehicle classes were defined for the simulation:

(1) Conventional vehicles: This type of vehicle has typical characteristics of a human-driven car. The default VISSIM car-following model (Wiedemann 74) was used. Furthermore, the uniform distribution with a minimum value of 45 km per hour and the maximum value of 55 km per hour was utilised to generate the speed of conventional vehicles.
(2) Connected and automated vehicles (CAVs): driving behaviour for this vehicle class consists of two major components, autonomous behaviour and connected behaviour, which will be explained below.

#### 3.3.1. Autonomous Behaviour

**Figure 3:**
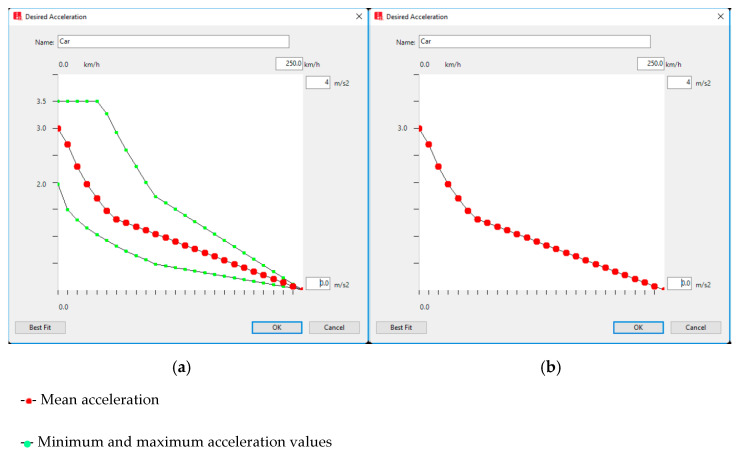
Desired acceleration functions of a conventional vehicle and an autonomous vehicle in VISSIM [58]. (**a**) Conventional vehicle; (**b**) Autonomous vehicle.

Numerous studies have investigated the characteristics of autonomous vehicles (AVs) through various experiments [53,54,55]. AVs have some common features which should be considered for the simulation. For instance, AVs can accept a smaller headway than conventional cars [53,55]. They can also keep their speed constant without any fluctuation during free-flow [56], so the constant speed of 50 km per hour was considered for CAVs in this research, which is the mean speed of conventional vehicles. Furthermore, autonomous vehicles accelerate more smoothly than conventional cars. Unlike conventional cars, every particular speed has a unique acceleration and deceleration rate for this type of vehicle, whose acceleration ranges between the minimum and maximum values [57]. Figure 3 better depicts the last cases.

In this study, the default AV behaviour of VISSIM, which is based on a European project called Coexist [59], was utilised. Coexist has tested its cars with four driving patterns (Rail Safe, Cautious, Normal, and All-Knowing). The main difference between these types of autonomous vehicles is their capability to accept headways. Cautious AVs are more conservative than conventional cars. Thus, they keep larger headways than other AV types. Cautious AVs were selected for simulating the autonomous behaviour of CAVs in this paper because this vehicle class may be the first generation of highly automated vehicles and can penetrate in transportation networks sooner than other AV types.

#### 3.3.2. Connected Behaviour

CAVs continuously pay attention to the data transmitted to them from other vehicles (V2V) and traffic infrastructures (V2I). In particular, CAVs receive information about the upcoming signal and modify their speed to arrive at the green phase without stopping in signalised intersections. Therefore, it is essential to use the internal script which reads this information from PTV VISSIM [58] and updates the desired speed of CAVs to arrive within green at the signal. This speed guidance is a rule-based algorithm [34] that developed mainly includes the following steps.

- Step 1. The first question that should be asked of all vehicles entering the network is if the car is able to receive signal data or not. Therefore, conventional vehicles will proceed with movement at their desired speed (the speed at which the driver wants to drive). However, if the vehicle is connected, Step 2 is executed.
- Step 2. The vehicle will continue with its current speed if it passes the intersection or no signal controller can be found ahead of this vehicle; otherwise, Step 3 must be performed.
- Step 3. In this step, the following question should be answered. “Is the signal at its green phase?”. If the response is negative and the signal controller is at its red phase, the vehicle speed must be adjusted (5). Otherwise, go to Step 4.
  V_opt_ = max(min(V_max for green start_, V_des_) − V_diff_, V_min_) (5)

In this equation, V_opt_ is the optimal speed of the vehicle, and V_des_ is the desired speed of the vehicle. Moreover, the functionality of V_diff_ is to adjust the vehicle speed so that the vehicle arrives shortly before the signal head. It was assumed to be 2 km per hour in the simulation. V_min_ is the least feasible speed of the vehicles in the network, which was considered 5 km per hour in this paper. Finally, the vehicle should not drive above the V_max for a green start_ in order to arrive just when the next green starts. This speed can be obtained from (6).

(6) $$V_{\max\;\mathrm{for}\;\mathrm{green}\;\mathrm{start}\;}=\frac{\mathrm{Vehicle}\;\mathrm{distance}\;\mathrm{to}\;\mathrm{signal}\;\mathrm{head}}{\mathrm{Time}\;\mathrm{until}\;\mathrm{the}\;\mathrm{next}\;\mathrm{green}\;\mathrm{phase}\;\mathrm{starts}}$$

- Step 4. If V_min for a green end_ (a minimum speed required to arrive at the intersection during the current green) is lower than the desired speed of the vehicle, then V_opt_ should be equal to V_des_. Conversely, Step 5 is executed. V_min for a green end_ can be calculated by (7).
  (7) $$V_{\min\;\mathrm{for}\;\mathrm{green}\;\mathrm{end}\;}=\frac{\mathrm{Vehicle}\;\mathrm{distance}\;\mathrm{to}\;\mathrm{signal}\;\mathrm{head}}{\mathrm{Time}\;\mathrm{until}\;\mathrm{the}\;\mathrm{next}\;\mathrm{red}\;\mathrm{phase}\;\mathrm{starts}}$$
- Step 5. If V_max for a green start_ is greater than the desired speed of the vehicle, then V_opt_ should be equal to V_des_. Otherwise, V_opt_ = V_max for a green start_. Therefore, the optimal speed of all CAVs in the network can be calculated through this procedure.

The proposed behaviours were entered in VISSIM for both conventional vehicles and CAVs. Now, the designated scenarios for this study should be explained. 

### 3.4. Simulation Scenarios

It is important to perform sensitivity analysis on various parameters to achieve more accurate and comprehensive findings. Therefore, several simulation scenarios were defined in this paper. Different market penetration rates of 0%, 25%, 50%, 75%, and 100% for CAVs were the first set of scenarios considered for this study. Moreover, three scenarios for the total demand were designated. The first scenario was called saturated, which has the travel demands equal to the capacity for each signal phase. This capacity can be calculated by (8). The second and third scenarios were called oversaturated and unsaturated, with travel demands equal to 1.1 and 0.7 of saturated conditions, respectively.

(8) $$Capacity=s\times \frac{g}{C}\times N$$

where *s* is the saturation flow rate was considered to be 1900 veh/h for each lane based on the Highway Capacity Manual [60]. Furthermore, *g* is the effective green time duration for the phase in seconds, *C* is the total cycle length in seconds, and *N* is the lane number for each lane group. 

Finally, different signal plans should be compared to measure the relative improvements of our approach compared to other widely used methods. First, the intersection with fixed signal timing alongside the AVs was investigated. This scenario assumes that vehicles cannot receive any information from the signal controller (AV scenarios do not include speed guidance approaches). This situation was called “Fixed AV”. Furthermore, the fixed signal timing was considered for the intersection again, but AVs, in this case, have the ability to receive signal data, and they can adjust their speed based on the speed guidance approach (i.e., CAVs were used for this condition instead of simple AVs). This situation was called “Fixed CAV”. The actuated signal plan was examined with different AV penetration rate scenarios (Not CAV). This case was named “Actuated”.

Thus, 60 scenarios (five different vehicle compositions × three different demands × four controlling conditions: Fixed AV, Fixed CAV, Actuated (AV), and RL (CAV)) were generated. Each scenario was typically executed in VISSIM for up to five various random seeds. This process was also accomplished by the RL, and the expected improvements in performance measures were assessed under the CAV environment.

## 4. Results

Two different performance measures are implemented: minimising total queue length and total stop delays. The main purpose of this paper is to develop a framework to minimise the performance measure. The minimisation of queue length is the first part of the performance index (PI). Queues happen when vehicles cannot pass the intersection at their green share, and they must stop behind the red light. The average queue length of all entries in the whole simulation period is assessed to optimise an RL feedback loop as a reward factor. The second part of PI is the stop delay of vehicles, a common performance measure in an optimal speed guidance system under a connected environment [28]. This can be described as a delay (in seconds), the mean stop time of vehicles waiting at the intersection behind red lights. In order to use the two aforementioned performance measures simultaneously in one expression, the transformation strategy has been used to convert each measure into a non-dimensional term. According to [61], the average queue length (*q*) can be divided by its maximum value (*Qmax*).

Moreover, stop delay (*d*) can be investigated per cycle length, so it should be divided by signal cycle time (*C*) discussed in the previous section. Therefore, (9) to (11) illustrate how to determine the PI value for each scenario. In these equations, *n* represents different replications for the simulation.

(9) $$\mathrm{Queue}\;\mathrm{Length}\;\mathrm{ratio}\;=\frac{1}{n}\;\sum_{i=1}^{n}\frac{q_{i}}{Qmax_{i}}$$

(10) $$\mathrm{Stop}\;\mathrm{Delay}\;\mathrm{ratio}\;=\frac{1}{n}\;\sum_{i=1}^{n}\frac{d_{i}}{C}$$

PI = Queue Length ratio + Stop Delay ratio(11)

**Figure 4:**
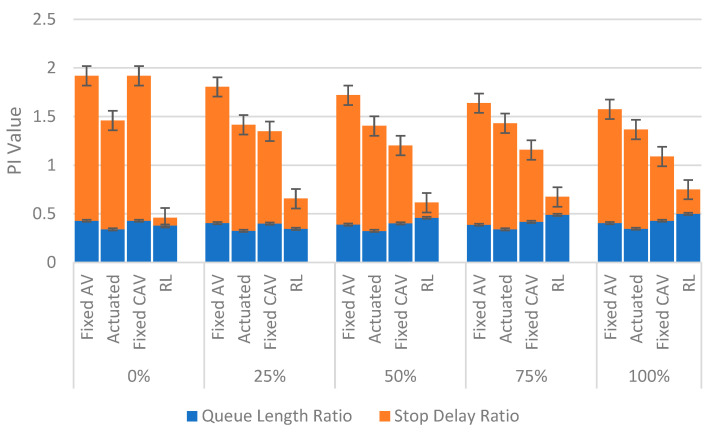
Comparing performance index values of the saturated scenarios.

**Figure 5:**
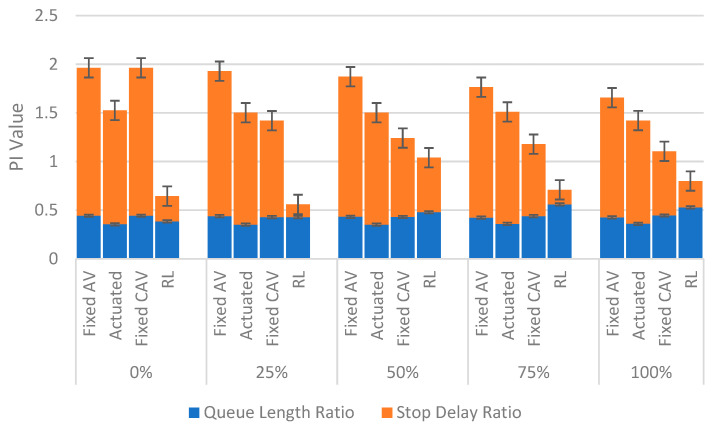
Comparing performance index values of the oversaturated scenarios.

In the first step, it is worth comparing PI values of various signal plans for both saturated and oversaturated conditions. The results for the average PI value are shown in Figure 4 and Figure 5.

As can be observed, the speed guidance approach has worked appropriately due to the fact that PI values for the fixed CAV condition are less than their corresponding values for the fixed AV condition in each scenario. In other words, a 100% penetration rate of CAVs compared to a conventional environment (0% penetration rate of CAVs) in the saturated scenario can decrease PI by 18% and 43% for fixed AV and fixed CAV conditions, respectively. Moreover, the fixed CAV situation has performed better than the actuated case. Therefore, the fixed CAV condition is the best case among all non-learning approaches, and the RL should be compared with this condition in order to assess its functionality.

As shown, the RL can noticeably reduce the PI compared to the fixed CAV condition, especially in 0% and 25% scenarios. However, a slight improvement has occurred in the last three vehicle composition scenarios (i.e., 50%, 75% and 100%), particularly for the oversaturated scenario. This is mainly because of the assumption that CAVs in this paper only can obtain the signal data. This assumption makes the simulation more straightforward and more rapid. Results show that the total network performance could be improved under this simple CAVs environment. Furthermore, future works should consider the CAVs that can receive and interpret the information transmitted from other CAVs (V2V). In other words, the driving behaviour implemented in this study for CAVs only can model V2I, and it cannot cover the V2V connection whose impact has been pinpointed from the 50% penetration rate for CAVs. 

Another point to note is that in most cases, the queue length ratio increases when the CAVs penetration rate also increases for the RL. For instance, the maximum value of the queue length ratio for the RL in the saturated scenario has a growth rate of 43% compared to its minimum value in the 25% scenario. Although it seems far from the expectation at first glance, it may stem from the fact that the speed guidance approach defined for this research does not consider vehicle positions, and CAVs receive the signal data as soon as they enter the network. Moreover, this algorithm does not specify different ranges for the minimum speed of CAVs, and it has been assumed to be 5 km per hour for this study. Therefore, such a wide range for receiving the signal information in conjunction with this tiny speed value can cause long queues behind CAVs. 

**Figure 6:**
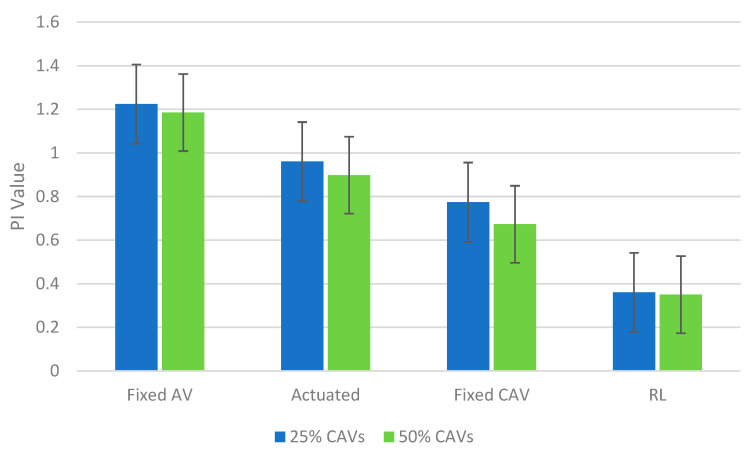
Comparing performance index values of the unsaturated scenario for all signal planning approaches.

PI values were analysed for both saturated and oversaturated scenarios. One of the unforeseen outcomes which can be highlighted in Figure 4 is that the PI value suddenly escalates when moving from 25% to 50% for the RL. In order to better perceive this trend, we present PI variation in the unsaturated condition for these two aforementioned CAV scenarios. The final results are displayed in Figure 6.

As expected, fixed CAV, actuated, and RL approaches can improve the conditions of this intersection compared to the simple fixed AV situation. The average improvements are 23%, 40%, and 70%, respectively. Therefore, RL has the best performance in unsaturated conditions among all other algorithms. The main reason for this is that despite using optimal fixed time for CAVs speed guidance, the proposed real-time RL could optimise the signal plan based on the time-varying traffic demand. This means that more vehicles can pass the intersection in their green time, and each entry will be vacant at its green share. That is why the queue length and stop delay will be minimised, and the intersection will perform at the ideal condition, which is unrivalled. 

Furthermore, all approaches show a decreasing PI rate by transitioning from 25% CAVs to 50% CAVs. Hence, it is concluded that, unlike saturated and oversaturated conditions, vehicle automation can play an apt role in unsaturated traffic conditions.

The results of this analysis also highlighted the fact that the PI value for a proposed RL framework with speed guidance under a CAV environment is much less than other control systems at all penetration rates of CAVs (AVs) and demand scenarios. The proposed framework is a novel way of framing the RL state based on a CAV environment with speed guidance that could work more flexibly than RL under a conventional environment or speed guidance system under CAVs with fixed timing signal control.

## 5. Conclusions

This study develops a learning-based framework that optimises the signal plan by training a traffic signal control as an agent in RL to minimise total queue length (reward in RL) when CAVs receive speed guidance in a fixed-time strategy to minimise the total stop delays. The signal controller (agent) was trained in the fully dynamic traffic environment (traffic flows and CAV speeds) under different demand levels (unsaturated, saturated, oversaturated) as well as CAV penetration rates (0%, 25%, 50%, 75%, 100%) scenarios to show potential interaction effects between signal timing plan, CAV penetration rate and traffic flow. 

An isolated four-leg signalised intersection with three phases (training example in VISSIM as a template of vehicle actuated signal control) was modelled to test the performance. The results were compared to a well-tuned fixed timing plan (with optimal speed advisory in a CAV environment) and actuated signal control. Two objective functions are implemented in a performance index (queue length and stop delay). An important finding from this study is that the proposed framework reduced stop delay significantly under all scenarios and was comparable to other control strategies in queue length.

Finally, the proposed framework only considers simple V2I communication in an isolated intersection. The framework is designed to handle urban networks, and the interaction in operations between adjacent intersections (for example, interchanges) under the V2X environment and evaluation for these conditions is planned. The main limitation of a network-wide application is the computational time for the RL training. In future works, offset optimisation could be added to the signal timing optimisation to implement the proposed approach in an arterial corridor with multiple traffic signals or a city traffic network. It can improve the overall performance of the proposed approach but increase the training process complexity for the RL approaches. Future research will also make use of V2V communication and dynamic speed guidance strategies. Lastly, with dynamic speed guidance strategies which have different communication and control levels compared to simple V2I, it might be helpful to analyse reward and state options of RL with different performance measures.

## Data Availability

The data are not publicly available due to the project requirements.

## References

[1] Institute of Economic Affairs (IEA) Discussion Paper, No.68, 2016. SEEING RED Traffic Controls and the Economy. https://iea.org.uk/wpcontent/uploads/2016/07/IEA%20Seeing%20Red%20%20Traffic%20Controls%20and%20the%20Economy.pdf.

[2] Gartner N.H. (1983). OPAC: A demand-responsive strategy for traffic signal control. Transp. Res. Rec. J. Transp. Res. Board.

[3] Head K.L., Mirchandani P.B., Sheppard D. (1992). Hierarchical framework for real-time traffic control. Transp. Res. Rec..

[4] Luyanda F., Gettman D., Head L., Shelby S., Bullock D., Mirchandani P. (2003). ACS-Lite algorithmic architecture: Applying adaptive control system technology to closed-loop traffic signal control systems. Transp. Res. Record.

[5] Mirchandani P., Head L. (2001). A real-time traffic signal control system: Architecture, algorithms, and analysis. Transp. Res. Part C Emerg. Technol..

[6] Ran Q., Yang J. A novel closed-loop feedback traffic signal control strategy at an isolated intersection. Proceedings of the 2012 IEEE International Conference on Information Science and Technology, ICIST.

[7] Fagnant D.J., Kockelman K. (2015). Preparing a nation for autonomous vehicles: Opportunities, barriers and policy recommendations. Transp. Res. Part A Policy Pract..

[8] Kockelman K., Boyles S. (2018). Smart Transport for Cities & Nations: The Rise of Self-Driving & Connected Vehicles. https://bit.ly/3gDpEaa.

[9] Elliott D., Keen W., Miao L. (2019). Recent advances in connected and automated vehicles. J. Traffic Transp. Eng..

[10] Feng Y., Head K.L., Khoshmagham S., Zamanipour M. (2015). A real-time adaptive signal control in a connected vehicle environment. Transp. Res. Part C Emerg. Technol..

[11] Yao H., Jin Y., Jiang H., Hu L., Jiang Y. (2022). CTM-based traffic signal optimisation of mixed traffic flow with connected automated vehicles and human-driven vehicles. Phys. A: Stat. Mech. Its Appl..

[12] Yao Z., Shen L., Liu R., Jiang Y., Yang X. (2020). A Dynamic Predictive Traffic Signal Control Framework in a Cross-Sectional Vehicle Infrastructure Integration Environment. Proc. IEEE Trans. Intell. Transp. Syst..

[13] Saldivar-Carranza E., Li H., Mathew J., Fisher C., Bullock D. (2022). Signalized Corridor Timing Plan Change Assessment Using Connected Vehicle Data. J. Transp. Technol..

[14] Yao Z., Jiang Y., Zhao B., Luo X., Peng B. (2020). A dynamic optimization method for adaptive signal control in a connected vehicle environment, Journal of Intelligent Transportation. Systems.

[15] Guo Q., Li L., Ban X. (2019). Urban traffic signal control with connected and automated vehicles: A survey. Transp. Res. Part C Emerg. Technol..

[16] Mintsis E., Vlahogianni E.I., Mitsakis E., Ozkul S. (2021). Enhanced speed advice for connected vehicles in the proximity of signalized intersections. Eur. Transp. Res. Rev..

[17] Jiang Y., Zhao B., Liu M., Yao Z. (2021). A Two-Level Model for Traffic Signal Timing and Trajectories Planning of Multiple CAVs in a Random Environment. J. Adv. Transp..

[18] He X., Liu H.X., Liu X. (2015). Optimal vehicle speed trajectory on a signalised arterial with consideration of queue. Transp. Res. Part C Emerg. Technol..

[19] Wang M., Daamen W., Hoogendoorn S.P., van Arem B. (2014). Rolling horizon control framework for driver assistance systems. Part I: Mathematical formulation and non-cooperative systems. Transp. Res. Part C Emerg. Technol..

[20] Wu X., He X., Yu G., Harmandayan A., Wang Y. (2015). Energy-optimal speed control for electric vehicles on signalised arterials. IEEE Trans. Intell. Transp. Syst..

[21] Wang P., Chan C.Y., de La F.A. A reinforcement learning-based approach for automated lane change maneuvers. Proceedings of the 2018 IEEE Intelligent Vehicles Symposium (IV).

[22] Abdulhai B., Kattan L. (2003). Reinforcement learning: Introduction to theory and potential for transport applications. Can. J. Civ. Eng..

[23] Shatnawi I., Yi P., Khliefat I. (2018). Automated intersection delay estimation using the input-output principle and turning movement data. Int. J. Transp. Sci. Technol..

[24] (2010). Traffic Analysis Toolbox Volume III: Guidelines for Applying Traffic Microsimulation Modeling Software. https://ops.fhwa.dot.gov/trafficanalysistools/tat_vol3/vol3_guidelines.pdf.

[25] Hunt P.B., Robertson D.I., Bretherton R.D. (1982). The SCOOT online traffic signal optimisation technique (Glasgow). Traffic Eng. Control..

[26] Sims A.G., Dobinson K.W. (1980). The Sydney Coordinated Adaptive Traffic (SCAT) System Philosophy and Benefits. IEEE Trans. Veh. Technol..

[27] Athmaraman N., Soundararajan S. Adaptive predictive traffic timer control algorithm. Proceedings of the 2005 Mid-Continent Transportation Research Symposium.

[28] Wang Y., Yang X., Liang H., Liu Y. (2018). A Review of the Self-Adaptive Traffic Signal Control System Based on Future Traffic Environment. J. Adv. Transp..

[29] Ke-Zhao B., Rui-Xiong C., Mu-ren L., Ling-Jiang K., Rong-sen Z. (2009). Study of the grade roundabout crossing. Comput. Sci..

[30] El-tantawy S., Member S., Abdulhai B., Abdelgawad H. (2013). Multiagent Reinforcement Learning for Integrated Network of Adaptive Traffic Signal Controllers Application on Downtown Toronto. IEEE Trans. Intell. Transp. Syst..

[31] Jing P., Huang H., Chen L. (2017). An Adaptive Traffic Signal Control in a Connected Vehicle Environment: A Systematic Review. Information.

[32] Wu W., Huang L., Du R. (2020). Simultaneous Optimization of Vehicle Arrival Time and Signal Timings within a Connected Vehicle Environment. Sensors.

[33] Katsaros K., Kernchen R., Dianati M., Rieck D. Performance study of a Green Light Optimized Speed Advisory (GLOSA) application using an integrated cooperative ITS simulation platform. Proceedings of the IWCMC 2011 7th International Wireless Communications and Mobile Computing Conference.

[34] Tang T.Q., Yi Z.Y., Zhang J., Wang T., Leng J.Q. (2018). A speed guidance strategy for multiple signalised intersections based on car-following model. Phys. A: Stat. Mech. Its Appl..

[35] Beak B., Larry Head K., Feng Y. (2017). Adaptive coordination based on connected vehicle technology. Transp. Res. Rec..

[36] Pandit K., Ghosal D., Zhang H.M., Chuah C.N. (2013). Adaptive traffic signal control with vehicular Ad Hoc networks. IEEE Trans. Veh. Technol..

[37] Yu C., Feng Y., Liu H.X., Ma W., Yang X. (2018). Integrated optimisation of traffic signals and vehicle trajectories at isolated urban intersections. Transp. Res. Part B Methodol..

[38] Lämmer S., Helbing D. (2008). Self-control of traffic lights and vehicle flows in urban road networks. J. Stat. Mech. Theory Exp..

[39] Carlson R.C., Papamichail I., Papageorgiou M. (2011). Local feedback-based mainstream traffic flow control on motorways using variable speed limits. IEEE Trans. Intell. Transp. Syst..

[40] Diakaki C., Papageorgiou M., Aboudolas K. (2002). A multivariable regulator approach to traffic-responsive network—Wide signal control. Control Eng. Pract..

[41] Chin Y.K., Lee L.K., Yang S.S., Tze K., Teo K. Exploring Q-Learning Optimisation in Traffic Signal Timing Plan Management. Proceedings of the 2011 Third International Conference on Computational Intelligence, Communication Systems and Networks.

[42] Chu T., Wang J., Codecà L., Li Z. (2019). Multi-agent deep reinforcement learning for large-scale traffic signal control. IEEE Trans. Intell. Transp. Syst..

[43] Kim D., Jeong O. (2019). Cooperative Traffic Signal Control with Traffic Flow Prediction in Multi-Intersection. Sensors.

[44] Yang K., Tan I., Menendez M. A reinforcement learning-based traffic signal control algorithm in a connected vehicle environment. Proceedings of the 17th Swiss Transport Research Conference (STRC 2017).

[45] Aslani M., Seipel S., Saadi M., Wiering M. (2018). Advanced Engineering Informatics Traffic signal optimisation through discrete and continuous reinforcement learning with robustness analysis in downtown Tehran. Adv. Eng. Inform..

[46] Prabuchandran K.J., Hemanth Kumar A.N., Bhatnagar S. Decentralised Learning for Traffic Signal Control. Proceedings of the Intelligent Transportation System Workshop, COMSNETS.

[47] Chen J., Xue Z., Fan D. (2020). Deep Reinforcement Learning Based Left-Turn Connected and Automated Vehicle Control at Signalized Intersection in Vehicle-to-Infrastructure Environment. Information.

[48] Liu W., Qin G., He Y., Jiang F. (2017). Distributed Cooperative Reinforcement Learning-Based Traffic Signal Control That Integrates V2X Networks’ Dynamic Clustering. Proc. IEEE Trans. Veh. Technol..

[49] Mousavi S.S., Schukat M., Howley E. (2017). Traffic light control using deep policy- gradient and value-function-based reinforcement learning. IET Intell. Transp. Syst..

[50] van der Pol E., Oliehoek F.A. Coordinated deep reinforcement learners for traffic light control. Proceedings of the 30th Conference on Neural Information Processing Systems.

[51] Nishi T., Otaki K., Hayakawa K., Yoshimura T. Traffic Signal Control Based on Reinforcement Learning with Graph Convolutional Neural Nets. Proceedings of the 2018 21st International Conference on Intelligent Transportation Systems (ITSC).

[52] Zhao J., Li W., Wang J., Ban X. (2015). Dynamic traffic signal timing optimisation strategy incorporating various vehicle fuel consumption characteristics. IEEE Trans. Veh. Technol..

[53] Aria E., Olstam J., Schwietering C. (2016). Investigation of Automated Vehicle Effects on Driver’s Behavior and Traffic Performance. Transp. Res. Procedia.

[54] ATKINS (2016). Research on the Impacts of Connected and Autonomous Vehicles (CAVs) on Traffic Flow.

[55] Stanek D., Huang E., Milam R.T. Measuring Autonomous Vehicle Impacts on Congested Networks Using Simulation. Proceedings of the Transportation Research Board 97th Annual Meeting.

[56] Asadi F.E., Anwar A.K., Miles J.C. Investigating the potential transportation impacts of connected and autonomous vehicles. Proceedings of the 2019 8th IEEE International Conference on Connected Vehicles and Expo, ICCVE.

[57] Zeidler V., Buck S., Kautzsch L., Vortisch P., Weyland C. Simulation of Autonomous Vehicles Based on Wiedemann’s Car Following Model in PTV Vissim. Proceedings of the Transportation Research Board 98th Annual Meeting.

[58] PTV Group PTV Vissim 10 User Manual. Ptv Ag. https://usermanual.wiki/Document/Vissim20102020Manual.1098038624.pdf.

[59] CoEXist (2020). Working towards a Shared Road Network. https://www.h2020-coexist.eu/.

[60] Council N.R. (2000). TRB. Highway Capacity Manual.

[61] Marler R.T., Arora J.S. (2005). Function-transformation methods for multi-objective optimisation. Eng. Optim..

